# Using Normative Language When Describing Scientific Findings: Randomized Controlled Trial of Effects on Trust and Credibility

**DOI:** 10.2196/45482

**Published:** 2023-03-30

**Authors:** Jon Agley, Yunyu Xiao, Esi E Thompson, Lilian Golzarri-Arroyo

**Affiliations:** 1 Prevention Insights Department of Applied Health Science, School of Public Health - Bloomington Indiana University Bloomington Bloomington, IN United States; 2 Department of Population Health Sciences Weill Cornell Medical College New York, NY United States; 3 The Media School Indiana University Bloomington Bloomington, IN United States; 4 Biostatistics Consulting Center School of Public Health - Bloomington Indiana University Bloomington Bloomington, IN United States

**Keywords:** trust, trust in science, scientific communication, meta-science, RCT

## Abstract

**Background:**

Scientists often make cognitive claims (eg, the results of their work) and normative claims (eg, what should be done based on those results). Yet, these types of statements contain very different information and implications. This randomized controlled trial sought to characterize the granular effects of using normative language in science communication.

**Objective:**

Our study examined whether viewing a social media post containing scientific claims about face masks for COVID-19 using both normative and cognitive language (intervention arm) would reduce perceptions of trust and credibility in science and scientists compared with an identical post using only cognitive language (control arm). We also examined whether effects were mediated by political orientation.

**Methods:**

This was a 2-arm, parallel group, randomized controlled trial. We aimed to recruit 1500 US adults (age 18+) from the Prolific platform who were representative of the US population census by cross sections of age, race/ethnicity, and gender. Participants were randomly assigned to view 1 of 2 images of a social media post about face masks to prevent COVID-19. The control image described the results of a real study (cognitive language), and the intervention image was identical, but also included recommendations from the same study about what people should do based on the results (normative language). Primary outcomes were trust in science and scientists (21-item scale) and 4 individual items related to trust and credibility; 9 additional covariates (eg, sociodemographics, political orientation) were measured and included in analyses.

**Results:**

From September 4, 2022, to September 6, 2022, 1526 individuals completed the study. For the sample as a whole (eg, without interaction terms), there was no evidence that a single exposure to normative language affected perceptions of trust or credibility in science or scientists. When including the interaction term (study arm × political orientation), there was some evidence of differential effects, such that individuals with liberal political orientation were more likely to trust scientific information from the social media post’s author if the post included normative language, and political conservatives were more likely to trust scientific information from the post’s author if the post included only cognitive language (β=0.05, 95% CI 0.00 to 0.10; *P*=.04).

**Conclusions:**

This study does not support the authors’ original hypotheses that single exposures to normative language can reduce perceptions of trust or credibility in science or scientists for all people. However, the secondary preregistered analyses indicate the possibility that political orientation may differentially mediate the effect of normative and cognitive language from scientists on people’s perceptions. We do not submit this paper as definitive evidence thereof but do believe that there is sufficient evidence to support additional research into this topic, which may have implications for effective scientific communication.

**Trial Registration:**

OSF Registries osf.io/kb3yh; https://osf.io/kb3yh

**International Registered Report Identifier (IRRID):**

RR2-10.2196/41747

## Introduction

### Background

The COVID-19 “infodemic” [1] has served as a stark reminder of the complexities of how people communicate and understand scientific information [2,3], and the importance of studying such information exchanges during public health crises [4]. “When people are presented with a high volume of information of varying accuracy, we propose that there exists a broad social interest in *people making decisions based on the best available evidence*” [5]. To that end, we endorse 2 premises: (1) the scientific method can produce knowledge about the world, and (2) no one can independently generate empirical knowledge about everything [6]. Thus, to make decisions based on the best available evidence, we must sometimes “trust what others tell us,” [7] because we cannot always generate such knowledge ourselves. Taken together, these premises also suggest that it is important to study factors that might influence trust in science and scientists as well as perceptions of their credibility. Indeed, a wide variety of research studies have demonstrated associations between trust in science and COVID-19 preventive behaviors and behavioral intentions [8-17].

However, it is also important not to oversimplify such concepts (eg, “trust the science”) [18]. Not everything that purports to be science has been derived from scientific procedures. Further, such simplification fails to acknowledge issues such as the degree of uncertainty in each empirical claim, the trustworthiness of a given scientist, or the rigor and reproducibility of the work underlying a claim. In addition, perceptions of trust and credibility may not *solely* rely on scientists’ epistemic (cognitive) claims. In our protocol [5] for this study, we outline our reasons for separating “cognitive” claims (eg, statements about *what is likely true of reality*) from “normative” claims (eg, recommendations about *what should be done* given certain information) [19].

Scientists often share cognitive claims (eg, the results of their work) *and* normative claims (eg, what should be done based on those results). Yet, it is entirely possible to trust a cognitive claim—to believe that something is true of the world—without trusting or relying on an associated recommendation about what should be done [20]. A fundamental difference is that normative claims make presumptions around *interests* [20]. For example, I may believe a cognitive claim that “it is safe to jump into a certain pool of water.” Yet, when I am told that I *should* jump in, a presumption is made that my primary hesitancy (ie, interest in not jumping in) is safety, and not a separate overriding concern (eg, perhaps I have a cell phone in my pocket). Studies have not generally used this exact linguistic framing, but we note that in a recent study in Germany conducted before and during the COVID-19 pandemic, the authors described their evidence as generally indicating that trust in scientists was largely predicated on their expertise, whereas distrust came from “violations of expectations regarding benevolence” [21]. Here, we infer that the idea of “benevolence” is less likely to apply to a cognitive claim (X is dangerous) and more likely to pertain to a normative claim (because X is dangerous, people should do Y), especially where “Y” is something that is seen as violating a person’s interests. Thus, in that specific example, trust appears to have been engendered in response to cognitive claims (ie, expertise) and distrust appears to have manifested in response to normative claims, where such claims highlighted discordant perceptions of interests between laypersons and scientists.

In further support of investigating differences between cognitive and normative claims, and their impact on perceptions of science and scientists, we point to evidence that greater belief in science may be associated with moralization of COVID-19 mitigation measures (eg, endorsing statements such as “Overall, I believe that *not* following C19 science recommendations is immoral”) [22]. Granted, “belief in science” is somewhat different from “trust in science.” Yet, we infer that assertions of morality or immorality are predicated on *what people believe should be done* rather than on empirical information. In other words, findings by Graso et al [22] suggested that it is plausible that a high degree of belief in science is associated with judgment of whether others *follow recommendations* (eg, normative claims), but presumably, this judgment would not logically apply to statements of fact (eg, cognitive claims).

### Study Objectives and Hypotheses

This study is designed to better understand the degree to which the use of cognitive and normative language by scientists influences perceptions of trust and credibility. Specifically, this paper reports the results from a highly granular, causal assessment of the effect of a single exposure to normative language from a scientist. For transparency, we use much of the language from our study protocol verbatim [5].

Our study will draw conclusions by randomizing a large, nationally representative sample of US adults to view a sample social media post that either (1) shares a cognitive claim from a 2020 study on face masks (control group), or (2) shares the same cognitive claim but also includes a normative claim about what people should do, given the cognitive claim, which is also from that study (intervention group; see the “Methods” section). We hypothesize the following:

- Hypothesis 1: Overall trust in science and scientists [23] will be significantly lower in the intervention arm (cognitive and normative claims) than the control arm (cognitive claim only).
- Hypotheses 2-5: The *perceived* credibility of the scientist who conducted the study, credibility of the research, trust in the scientific information on the post, and trust in scientific information coming from the author of the post [24] will each be significantly lower in the intervention arm (cognitive and normative claims) than the control arm (cognitive claim only).
- Preregistered analyses (without hypotheses): We will study the interaction between the study arm and political orientation for each of the 5 preregistered hypotheses. We included these analyses because of complex and nuanced, but consistent evidence that trust in science and scientists is associated with political orientation [23,25-27]. However, we were uncertain whether and how political orientation might interact with the type of language used, so did not predict directionality.

## Methods

### Study Design and Participants

Participants were a nationally representative US sample by cross sections of age, sex, and race/ethnicity obtained using the online research and data collection platform Prolific (Prolific Academic Ltd). Prolific maintains a panel of around 130,000 research participants and verifies the identity of all accounts using a 3-part procedure including email, phone number, and identity, such as US driver’s license or passport [28]. The nationally representative US sample is drawn from a subset of those participants whose age, sex, and race/ethnicity cross-tabulation is proportional to the recent US Census [29]. To be eligible for the study, participants were also required to be aged 18 or older.

Before randomization, we implemented 4 quality control questions to attempt to limit the impact of automated, dishonest, or inattentive respondents [30]. An example of such an item (to check for dishonesty) is, “In the past 2 years, have you ever traveled to, or done any business with entities in, Latveria?” with the response options: No, never; Yes, but not in the past 2 years; Yes, within the past 2 years, where any response other than “No, never” was considered dishonest because Latveria only exists within the Marvel Comic Universe. Those items were intermixed with the study’s sociodemographic questions. Individuals who failed any quality control checks were considered ineligible for the study and asked to return the study to Prolific without applying for compensation. Those slots were then reselected in a manner preserving the representative nature of the sample.

All participants digitally provided informed consent prior to beginning the study (Indiana University Institutional Review Board, approval number 16141). Participants who successfully completed the study and submitted a compensation request to Prolific were paid US $1.50. Participants whose data were used took an average of 7.37 (SD 9.61) minutes to complete the study. This study was preregistered using the Open Science Framework and the protocol was published in full prior to any data collection [5].

### Randomization, Masking, and Enrollment

Participants who passed all quality control checks were randomized to 1 of the 2 study arms. The intervention arm (arm 1) contained an image of a social media post about face masks for reducing COVID-19 transmission that included both cognitive and normative claims. The control arm (arm 2) contained the same image except that it did not include the normative claim. Both images were otherwise identical.

Prolific fully managed enrollment. Individuals who were enrolled accessed a link that we provided to our study in the online study platform Qualtrics XM (Qualtrics International Inc). Participants who were not rejected by quality control were randomized using the Qualtrics “randomizer” tool, hence no study personnel were involved in assignment to arms. To avoid enrollment by individuals specifically interested in the study topic, our invitation summary text was vague (“We are interested in understanding how people perceive and think about messages”). Initial analyses were completed by a statistician (LGA) who was blinded to the meaning of the arm variable.

### Procedures

**Figure 1:**
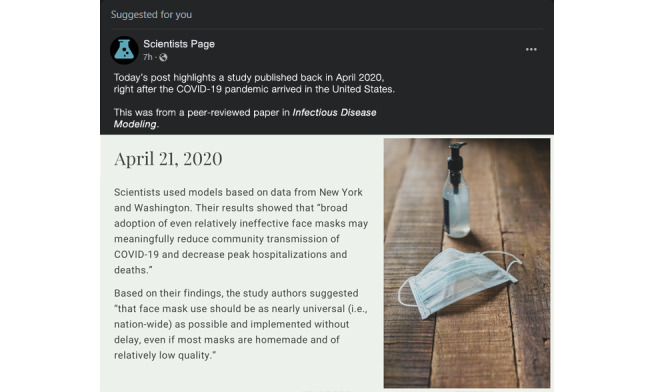
Intervention image.

**Figure 2:**
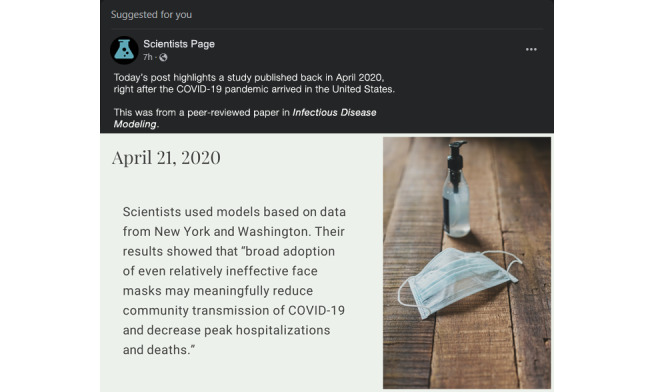
Control image.

As previously described, participants who began the study in Qualtrics completed sociodemographic questions intermixed with quality control checks (see our rationale for the importance of such checks in [30]). Eligible participants were randomized to a study arm (though this was not indicated to participants in any way). Participants were then shown a message reading, “Please view the following image. Take as much time as you need, but you will not be able to move forward for 30 seconds. After 30 seconds, the forward button will appear.” Below the message was either the intervention image (arm 1; Figure 1 and Multimedia Appendix 1) or the control image (arm 2; Figure 2). To ensure that the images were easily viewed, we used the Lightbox script [31] which allowed participants to enlarge and reduce the image size. As we specified in the protocol, we incorporated 2 mechanisms to improve ecological validity [32]. First, the social media posts were formatted based on the “suggested for you” post structure for Facebook, and second, the language used to illustrate cognitive and normative claims was actual language from a notable peer-reviewed study [33].

Once participants advanced, they were shown a smaller copy of the image they had just viewed along with 1 (control) or 2 (intervention) comprehension questions. Participants who responded correctly to all questions advanced in the study, and those who did not respond correctly were returned to the larger image and shown the message, “At least one of your answers was incorrect. Please re-read the post carefully and then answer the following (one/two) questions to verify your understanding of the post.” Such participants then moved forward to the comprehension question page again. The comprehension questions were prespecified in our protocol [5]:

- Both arms: (true/false) “In the social media post you read about a scientific study from April 2020, the study authors found that face masks could reduce the spread of COVID-19 as well as lowering hospitalizations and deaths.”
- Intervention arm only: (true/false) “In the social media post you read about a scientific study from April 2020, the study authors recommended that everyone in the US should start wearing masks immediately.”

Comprehension was generally good. For the intervention group, 38/778 (4.9%) responded incorrectly to 1 or both questions the first time, and only 5 participants did the second time (0.6%). In the control arm, 14/748 (1.9%) responded incorrectly to the question the first time, and none did the second time.

Finally, participants completed all prespecified outcome measures and covariates (see the “Outcomes” section and the protocol [5]) and the study concluded.

### Outcomes

There were 5 primary outcome variables, each of which corresponded to a hypothesis as well as a prespecified reanalysis of that hypothesis (in which arm × political orientation was added as an interaction term). The variables were collected as prespecified, so we directly copied the text from our protocol here [5].

The overall trust in science and scientists was measured by the 21-item scale developed and validated by Nadelson et al [23]. An example item from the scale measures agreement with the statement, “When scientists change their mind about a scientific idea it diminishes my trust in their work.” All items used the response options (1=strongly disagree; 2=disagree; 3=neutral; 4=agree; and 5=strongly agree), but some items were reverse coded. This scale has demonstrated excellent internal reliability in our previous studies with crowdsourced samples (α>.900) [25,34-37] and had excellent reliability with this sample (α=.954; n=1520). The final score represents the overall level of trust ranging from (1=low) to (5=high).

- Measures of credibility and trust that were specific to the hypothetical social media post and the scientist who conducted the study (from Song et al [24]) were as follows:
  - “How credible is the scientist who conducted the study described in the post?” (1=not credible at all to 7=extremely credible); note that this language is slightly different from the original item to avoid ambiguity arising from the potential that a scientist authored the social media post;
  - “How credible is this research?” (1=not credible at all to 7=extremely credible);
  - “I would trust scientific information if I knew it came from this author.” (1=strongly disagree to 7=strongly agree);
  - “I trust this scientific information.” (1=strongly disagree to 7=strongly agree).

### Covariates

As with the outcome variables, the covariates were collected as prespecified, so we directly copy the text from our protocol here [5].

- Familiarity with science was measured by 1 item asking, “How often do you read science papers or science in the news?” (1=never to 5=always); this item was suggested in Song et al [24] as being potentially important to consider when studying how people perceive science and scientists.
- Level of religious commitment (0=low to 10=high), as used in our previous studies [25,34,36]. Multiple studies have shown an association between level of religious commitment and trust in science and scientists (eg, [23,25,38,39]).
- Political orientation (0=liberal to 10=conservative), as used in our previous studies [25,34,36]. As described previously, studies have suggested that political orientation is associated with trust in science and scientists [23,25-27].
- Political party (Republican, Democrat, or other), given recent research suggesting divergence between political orientation and party orientation pertaining to face masks [40].
- Race, ethnicity, gender, age (“About how old are you [in years]?”), and education level (“What is the highest grade or level of school you have completed, or the highest degree you have received?”) from the PhenX Toolkit (RTI International) [41].

### Statistical Analysis

We planned to recruit 1500 participants, which would allow the detection of small effects at α=.05 (Cohen *d*=0.14) and at α=.01 (Cohen *d*=0.18) for between-group differences at 80% power [5]. We committed to reporting exact *P* values and to being cautious in our interpretations [42]. Missingness was low (no more than 0.6% by variable), so cases with missing data were treated using pairwise exclusion. Data were exported to and cleaned using SPSS version 28 (IBM Inc), and all subsequent analyses were conducted using R version 4.2.2 (R Foundation for Statistical Computing).

For hypotheses 1 through 5, we used analysis of covariance, with the study arm (intervention or control) as the independent variable and each of the outcome variables set as the dependent variable (1 per hypothesis). Each analysis incorporated all measured covariates and was checked for violations of any statistical assumptions prior to being run (no violations were found; see Multimedia Appendix 2). For the recalculated hypotheses 1 through 5 (with arm × political orientation included), we used linear regression incorporating the remaining covariates, and likewise verified the absence of statistical violations prior to completing the analyses (see Multimedia Appendix 2). All findings are reported using β values, 95% CIs of β, and *P* values. Other means of interpretation, such as *F* statistics and sums of squares, are available in Multimedia Appendix 2.

### Ethics Approval and Consent to Participate

All participants digitally provided informed consent prior to beginning the study (Indiana University Institutional Review Board, approval number 16141).

## Results

### Overview

Additional details from the study, including raw data, data cleaning syntax, and analytic code, are available in Multimedia Appendix 2. During the review process, we were also asked to run the analyses without covariates (eg, using only the study arm). These overall results were similar to the results for hypotheses 1 through 5 and the full output from those exploratory analyses is also available in Multimedia Appendix 2.

### Sample Characteristics

A total of 1635 unique individuals accepted the survey in their Prolific account and accessed the study between September 4, 2022, and September 6, 2022, when 1500 surveys were verified for payment. Prior to randomization, cases were removed from the data set and resampled if they declined to participate after reading the study information sheet (n=2) or agreed to the study information sheet but exited the survey immediately thereafter (n=6). Cases were also deleted prior to randomization and resampled based on the first missed quality control check (although some participants might have missed multiple checks, numbers here represent unique cases): dishonesty or misrepresentation (n=14) or inattention (first attention check, n=20; second attention check, n=48). Some individuals quit the survey prior to randomization but did not miss any attention checks (n=11) and were removed and resampled. Finally, several individuals were randomized and accessed the intervention but did not provide any study data (n=8). These cases were also eliminated from analysis, but their arm assignment was not adjusted.

**Figure 3:**
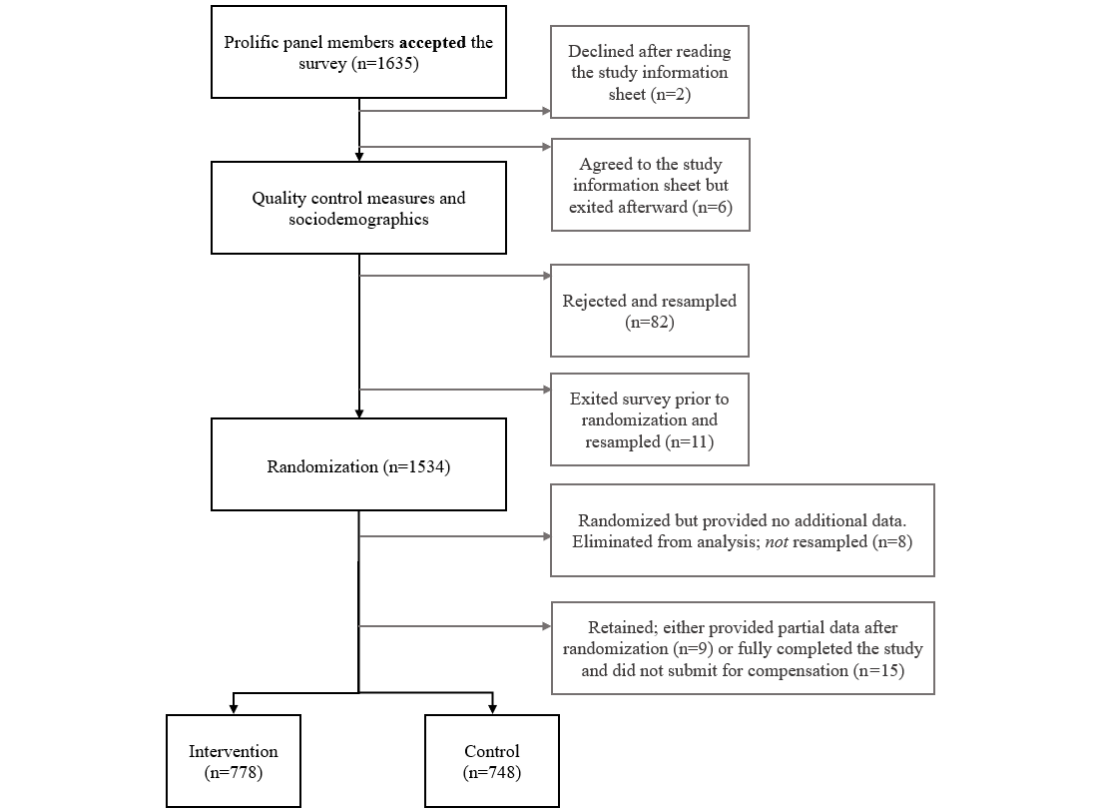
CONSORT (Consolidated Standards of Reporting Trials) study diagram.

A few individuals quit part way through the study but provided some data (n=9) after randomization; 15 individuals also fully completed the survey but did not submit a request for compensation. These individuals were retained in the arm to which they were assigned. Thus, the final total number of cases retained for analysis was 1526, of which 9 had partial data and 1517 had complete data, with 778 usable cases allocated to the intervention arm and 748 usable cases allocated to the control arm (Figure 3). Sample characteristics by study arm are provided in Table 1.

**Table 1:** Sample characteristics by study arm.

Variable			Intervention (n=778)		Control (n=748)
**Gender, n (%)**					
	Male	354 (45.5)		378 (50.5)	
	Female	416 (53.5)		361 (48.3)	
	Nonbinary	7 (0.9)		9 (1.2)	
	Transgender	1 (0.1)		0 (0.0)	
**Race, n (%)**					
	White	626 (80.5)		567 (75.8)	
	Black or African American	99 (12.7)		108 (14.4)	
	American Indian or Alaska Native	3 (0.4)		1 (0.1)	
	Asian	40 (5.1)		54 (7.2)	
	Other	10 (1.3)		18 (2.4)	
Hispanic or Latino (Yes), n (%)			37 (4.8)		46 (6.1)
Age (years), mean (SD)			45.8 (16.3)		45.2 (15.8)
**Highest education, n (%)**					
	Less than high school (no diploma or general educational development)	9 (1.2)		7 (0.9)	
	High school graduate, general educational development, or equivalent	111 (14.3)		87 (11.6)	
	Some college, but no degree	167 (21.5)		163 (21.8)	
	Associate degree or bachelor’s degree	341 (43.8)		354 (47.3)	
	Master’s degree	104 (13.4)		106 (14.2)	
	Doctoral or professional school degree	46 (5.9)		31 (4.1)	
Religious commitment (1=low to 10=high), mean (SD)			3.94 (3.38)		3.76 (3.31)
Political orientation (1=liberal to 10=conservative), mean (SD)			4.07 (2.72)		4.11 (2.67)
Frequency reading science papers/news (1=never to 5=always)			2.93 (0.86)		2.95 (0.92)
**Political orientation, n (%)**					
	Republican	158 (20.3)		147 (19.7)	
	Democrat	422 (54.2)		405 (54.1)	
	Other	193 (24.8)		192 (25.7)	
Credibility of the scientist (1=not at all credible to 7=extremely credible), mean (SD)			4.96 (1.45)		4.87 (1.47)
Credibility of the research (1=not at all credible to 7=extremely credible), mean (SD)			4.98 (1.57)		4.93 (1.57)
Trust scientific information from this author (1=strongly disagree to 7=strongly agree), mean (SD)			4.74 (1.47)		4.70 (1.50)
Trust this scientific information (1=strongly disagree to 7=strongly agree), mean (SD)			5.11 (1.62)		5.03 (1.62)
Trust in science and scientists (1=low, 5=high), mean (SD)			3.72 (0.69)		3.72 (0.73)

### Hypothesis 1

**Figure 4:**
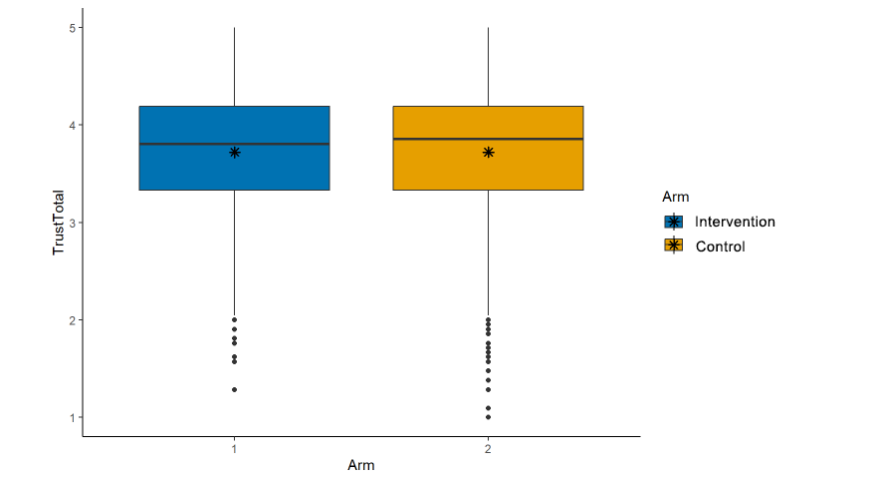
Main effects (arm, trust in science/scientists).

We hypothesized that overall trust in science and scientists [23] would be significantly lower in the intervention arm (cognitive plus normative language) than in the control arm (cognitive language alone). This hypothesis was not upheld, as we found no evidence of a difference between study arms (μ_int_=3.72; μ_control_=3.72; β=0.00, 95% CI –0.06 to 0.05; *P*=.89; Figure 4).

### Hypotheses 2 Through 5

**Figure 5:**
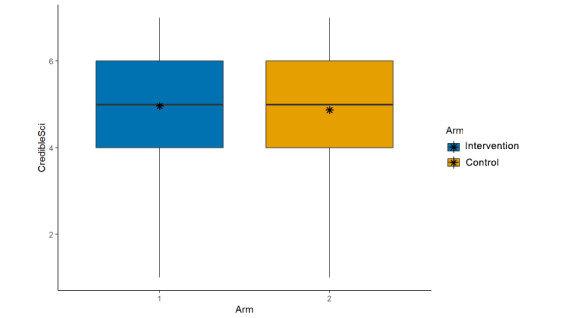
Main effects (arm, credibility of the scientist).

**Figure 6:**
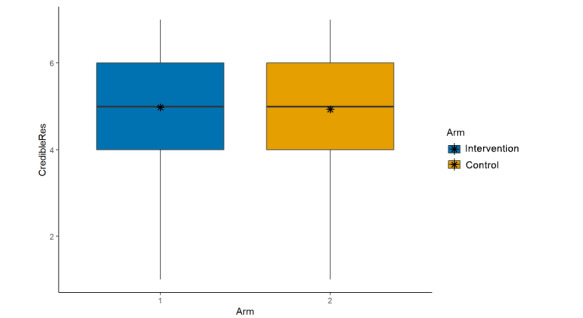
Main effects (arm, credibility of the research).

**Figure 7:**
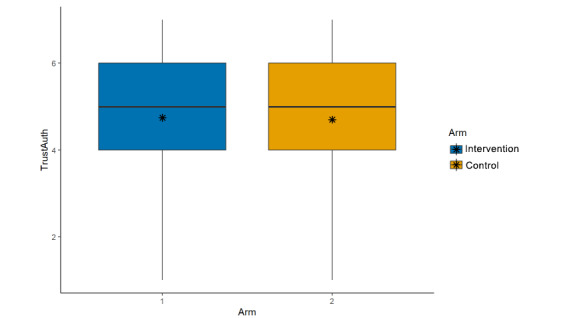
Main effects (arm, trust scientific information from this author).

**Figure 8:**
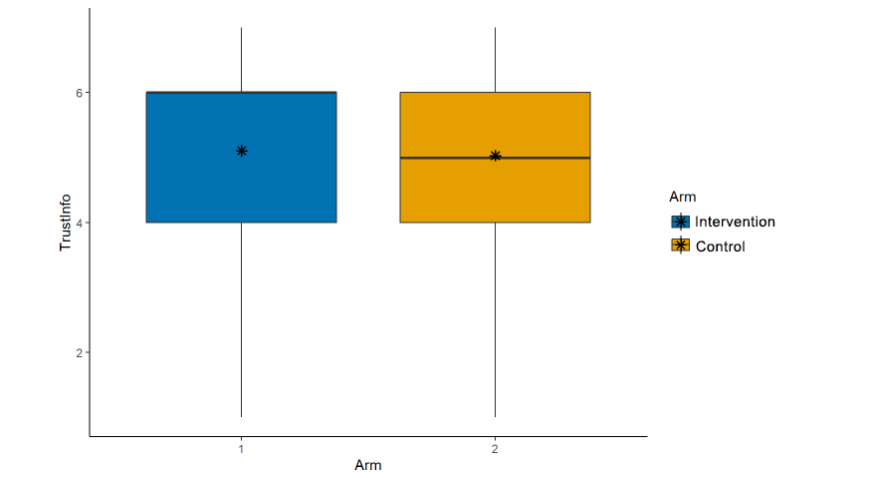
Main effects (arm, trust this scientific information).

We hypothesized that each of the 4 single-item measures of trust and credibility specific to the featured post and the scientist who conducted the study would be significantly lower in the intervention arm (cognitive plus normative language) than in the control arm (cognitive language alone) [24]. For the analyses of main effects, none of the hypotheses was upheld, as no significant differences were observed between study arms. This was true for:

- Credibility of the scientist who conducted the study described in the post (μ_int_=4.96; μ_control_=4.87; β=–0.08, 95% CI –0.21 to 0.06; *P*=.26; Figure 5);
- Credibility of the research described in the post (μ_int_=4.98; μ_control_=4.93; β=–0.03, 95% CI –0.17 to 0.12; *P*=.71; Figure 6);
- Trusting scientific information if it came from this author (μ_int_=4.74; μ_control_=4.70; β=–0.04, 95% CI –0.18 to 0.10; *P*=.57; Figure 7);
- Trusting this scientific information (μ_int_=5.11; μ_control_=5.03; β=–0.06, 95% CI –0.20 to 0.08; *P*=.42; Figure 8).

### Additional Preregistered Analyses

We preregistered additional analyses for this study focused on interactions between arm assignment and political orientation [5], but we did not include specific hypotheses because we did not have an inclination as to how the specified interactions might function. These investigations examined the same outcome variables as hypotheses 1 through 5 and used the same covariates, but also included an interaction term for political orientation × arm. Here, we focused specifically on the effects of study arm, political orientation × arm interaction, and political orientation. Other covariates were sometimes significant in models (see Multimedia Appendix 2).

### Study Arm

In 4 of the 5 additional analyses, the study arm remained nonsignificant, including for overall trust in science and scientists (β=0.01, 95% CI –0.10 to 0.11; *P*=.97), credibility of the scientist (β=–0.23, 95% CI –0.48 to 0.01; *P*=.06), credibility of the research (β=–0.18, 95% CI –0.44 to 0.08; *P*=.17), and trusting this scientific information (β=–0.20, 95% CI –0.45 to 0.06; *P*=.13). However, participants in the control arm (cognitive language only) reported lower trust in scientific information from the author (β=–0.26, 95% CI –0.50 to –0.01; *P*=.04).

### Arm × Political Orientation Interaction

**Figure 9:**
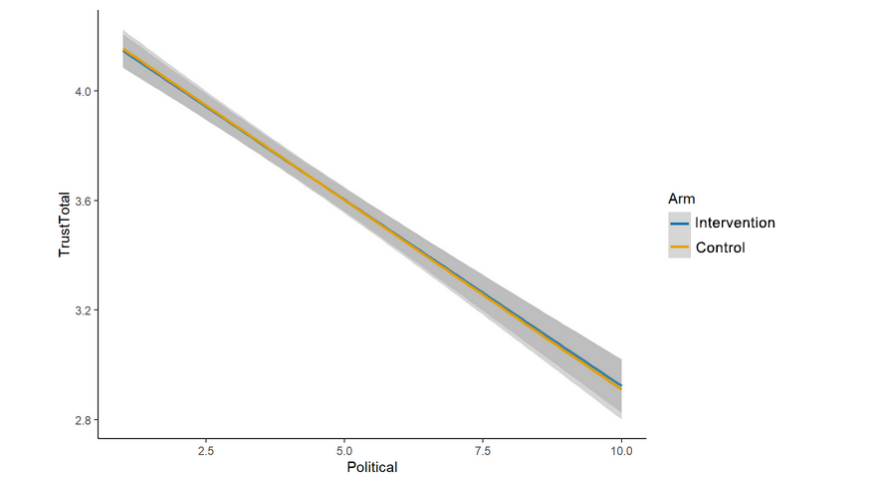
Interaction (arm × political orientation, trust in science/scientists).

**Figure 10:**
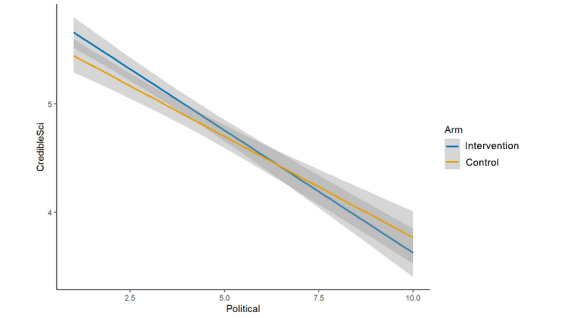
Interaction (arm × political orientation, credibility of the scientist).

**Figure 11:**
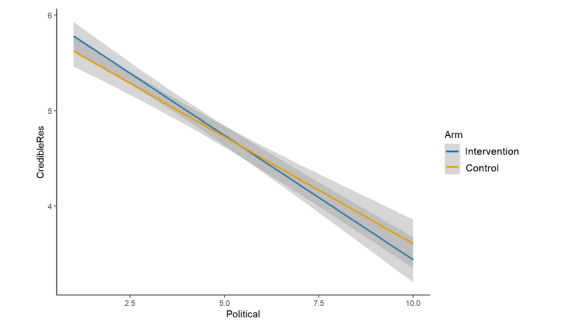
Interaction (arm × political orientation, credibility of the research).

**Figure 12:**
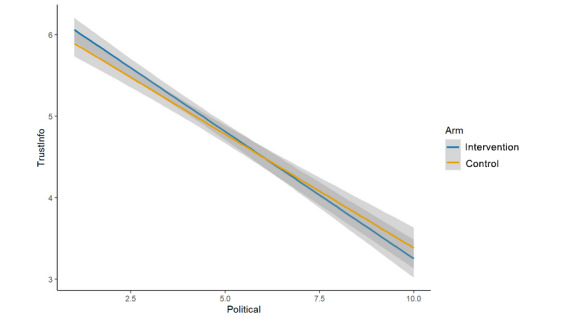
Interaction (arm × political orientation, trust this scientific information).

**Figure 13:**
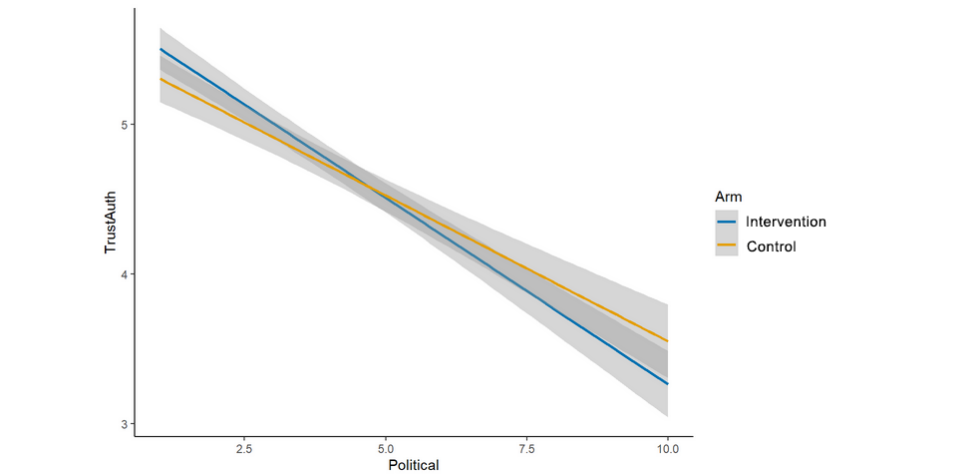
Interaction (arm × political orientation, trust scientific information from this author).

The interaction between study arm and political orientation was nonsignificant for overall trust in science and scientists (β=0.00, 95% CI –0.02 to 0.02; *P*=.89; Figure 9), credibility of the scientist (β=0.04, 95% CI –0.01 to 0.09; *P*=.13; Figure 10), credibility of the research (β=0.04, 95% CI –0.02 to 0.09; *P*=.17; Figure 11), and trusting this scientific information (β=0.03, 95% CI –0.02 to 0.09; *P*=.20; Figure 12). There was evidence of an interaction effect for trusting scientific information from the author; more liberal respondents were more likely to trust the intervention message’s author (cognitive plus normative language) and more conservative respondents were more likely to trust the control message’s author (cognitive language only; β=0.05, 95% CI 0.00 to 0.10; *P*=.04; Figure 13). In addition, visual inspection identified clearly intersecting lines in some cases of nonsignificant interactions, so it is possible, although we cannot determine from this study, that an interaction effect exists that is sufficiently small we were underpowered to test it.

### Political Orientation

Political orientation was strongly associated with each variable (more conservative orientation was associated with lower scores). This was true for overall trust in science and scientists (β=–0.13, 95% CI –0.15 to –0.12; *P*<.001), credibility of the scientist (β=–0.26, 95% CI –0.29 to –0.22; *P*<.001), credibility of the research (β=–0.29, 95% CI –0.33 to –0.25; *P*<.001), trust in scientific information from the author (β=–0.27, 95% CI –0.31 to –0.23; *P*<.001), and trusting this scientific information (β=–0.33, 95% CI –0.37 to –0.29; *P*<.001).

### Exploratory Results

This study was not intended to study the covariates in detail, but we would be remiss in failing to mention an interesting feature of both the basic models and the models with the arm × political interaction (all outcome tables are available in Multimedia Appendix 2). Specifically, the question intended to measure *familiarity with science*, “how often do you read science papers or science in the news,” was strongly and positively associated with all forms of trust and credibility.

## Discussion

### Principal Findings

We conducted a large, preregistered, randomized controlled trial to test whether the addition of normative language to cognitive language about face masks in otherwise identical social media posts influenced 5 different measures of trust and credibility of science and scientists. As we outlined in our “Introduction” section as well as in our protocol paper [5], we expected that reading normative language that made recommendations about wearing face masks would reduce the extent to which participants would find science/scientists trustworthy or credible (compared with participants who read cognitive language about the expected effects of face masks without the associated recommendations). However, it appears that we were wrong. Our first 5 hypotheses, which tested the effects of study arm (ie, intervention vs control) without any interactions, were not supported, suggesting that, in general, a single instance of a normative claim (even about a contentious issue such as face masks during COVID-19) did not have an effect on any of our measures of trust or credibility.

We also prespecified that we would conduct the same analyses while including an interaction term for study arm × political orientation. We made that decision because an association between political orientation and trust in science was observed in the validation and development study for the scale we used to measure overall trust in science and scientists (Nadelson et al [23]), in our own work (eg, [25]), and in multiple other studies, though with various nuances (eg, the type of science being queried) [27,43,44]. Because of the complexity of the association, we did not speculate as to how such an interaction might manifest in this experiment (eg, hypothesis generation) and instead merely indicated that we would conduct the analyses [5]. For the single-question variable *trust scientific information from this author*, both the effect of arm and the interaction effect of arm × political orientation were marginally significant (.01<*P*<.05), with political liberals being more likely to trust scientific information from authors who provided both cognitive and normative claims, and political conservatives more likely to trust scientific information from authors who provided only cognitive claims.

### Interpretation

This study examined the effects of normative language using very specific parameters: a single exposure to normative plus cognitive language from a study in comparison to cognitive language alone, at a dose of at least 30 seconds. Further, the exposure specifically pertained to a topic that is highly politicized in US culture (COVID-19 face masks). In those specific circumstances, *when treating the sample as a whole*, the introduction of normative language did not appear to have a meaningful effect on any of our outcome measures. We do not know how the results would manifest in the cases of repeated exposures or if presented using different scientific topics. We also note that it is plausible, though not by any means certain, that the effects of normative language on trust and credibility manifest slightly differently (in opposite directions) on the ends of the political spectrum.

At the same time, we note that political orientation had an extremely strong, negative association with our measures in this study, regardless of the study arm. We also note that except in the case of the aggregated measure (overall trust in science and scientists), there was a clear, visible intersection (Figures 10-13) of the study arms across the political spectrum, though with an overlap of the 95% CIs in many cases (and a significant interaction, as noted, for *trusting scientific information from this author*). However, except possibly in the specific instance of “scientific information from this author” and in the context of face masks for COVID-19, this study cannot determine whether these visible trends represent true, small differences or whether they are artifacts of unexplained variability.

The shape of the data does cause us to wonder whether the following questions might be important to study:

- Are there very small differences (eg, smaller than we were able to test in this study) between how political liberals and political conservatives perceive normative language, such that single exposures increase trust and credibility among liberals and reduce it among conservatives?
- If the answer to the above question is yes, do such effects matter individually, and is there a cumulative effect of such exposures, such that the very small effect sizes observed for single exposures are summative or multiplicative in impact over time?

Given the prima facie evidence that cognitive claims and normative claims are different, and the importance of understanding trust in science and scientists, and credibility thereof, we believe that additional probative studies (such as those described above) are important and may be useful. Debates about science communication are international in scope and can have high stakes. As an example, we point to a recently published postmortem review of Sweden’s COVID-19 policy, which lays out the complexity of scientific and policy interaction; although not its primary purpose, that document shows some ways in which cognitive and normative claims can be bundled together in high-stakes communication and decision making [45].

However, after considering the totality of our study results, we note that substantive shifts in perceived trust or credibility appear unlikely to manifest in response to *single* instances of normative language, even around highly contentious topics.

### Limitations and Conclusions

In addition to the caveats addressed throughout our manuscript, we note several additional considerations that are important when interpreting this work. First, although the study sample was nationally representative by cross sections of age, gender, and race/ethnicity, the Prolific service is an online program, which may raise some concerns about generalizability (eg, party affiliation is more often Democrat in our sample than in national Gallup polls [46]). However, as we note in our protocol, “Studies have found the Prolific platform to produce high-quality research data in general and relative to competing services (eg, Qualtrics and Dynata panels, Amazon Mechanical Turk, and CloudResearch)” [47,48]. We provided an analysis of the perceived limitations of Prolific in a separate protocol we published in 2020 [49], especially with regard to the representative sampling of age as a means of attenuating possible bias from the platform being online. Second, there are a number of theoretical assumptions embedded in the design of a study such as this one—we have attempted to be very clear and precise in terms of what we measured, and what we did not, but readers should be careful not to make sweeping conclusions. For example, the findings might be different for less contentious narrative topics. Third, although the analyses were preregistered and limited in number, there is still an inflated risk of type 1 error, which is why we use cautious language in interpreting some of our findings where 0.01<*P*<0.05. Finally, we note that the group sizes were somewhat uneven. The Qualtrics XM randomizer tool allows for “alternating” assignment (eg, 1, 2, 1, 2), but such assignment is not truly random. Thus, we instead set an option for true random assignment with the parameter that 750 people needed to be allocated to each arm at the end of the study. However, because of rejection, quality control procedures, and submissions not claiming payment, the distribution was uneven after data were cleaned. This possibility was anticipated in the protocol and should not substantively alter interpretation of the findings because the randomness of assignment was preserved.

## Appendix

Multimedia Appendix 1 CONSORT-eHEALTH checklist (V 1.6.1).

Multimedia Appendix 2 Data, code, and syntax.
