# Supplementary material for: Using Normative Language When Describing Scientific Findings: Randomized Controlled Trial of Effects on Trust and Credibility
Source: J Med Internet Res. 2023 Mar 30;25:e45482. doi: 10.2196/45482 (PMC10131812; doi:10.2196/45482)
Supplement: Multimedia Appendix 2 [file jmir_v25i1e45482_app2.zip › Normative-Cognitive-analysis-2023-03-05 (1).html]

Normative-Cognitive study


Code 

- Show All Code
- Hide All Code

# Normative-Cognitive study

#### Lilian Golzarri-Arroyo

#### 2023-03-05

```
# Read data
df <- haven::read_sav("../Data/N-C+Study_September+6,+2022_15.08 LGA clean.sav")

# Set levels
df$Gender <- haven::as_factor(df$Gender)
df$Race <- haven::as_factor(df$Race)
df$Ethnicity <- haven::as_factor(df$Ethnicity)
df$Arm <- haven::as_factor(df$Arm)
df$Educ <- haven::as_factor(df$Educ)


# Set all as factors
df_cat <- haven::as_factor(df)
```

From Jon’s email:

For the primary analyses, Covariates=Q57, Party, Political,
Religious, Gender, Race, Ethnicity, Age; Independent=Arm

- Hypothesis 1: ANCOVA, Dependent=TrustTotal
- Hypothesis 2: ANCOVA, Dependent=CredibleSci
- Hypothesis 3: ANCOVA, Dependent=CredibleRes
- Hypothesis 4: ANCOVA, Dependent=TrustAuth
- Hypothesis 5: ANCOVA, Dependent=TrustInfo

Preregistered Exploration: Repeating Hypotheses 1 through 5 with
Linear Regression to check the interaction term of Political\*Arm.

# Descriptive statistics

## Values as Continuous

```
table1(~Age+Gender+Race+Ethnicity+Educ+Q57+Party+Political+Religious+TrustTotal+CredibleSci+CredibleRes+TrustAuth+TrustInfo|Arm,data=df)
```

|  | 1 (N=778) | 2 (N=748) | Overall (N=1526) |
| --- | --- | --- | --- |
| About how old are you (in years)? |  |  |  |
| Mean (SD) | 45.8 (16.3) | 45.2 (15.8) | 45.5 (16.1) |
| Median [Min, Max] | 46.0 [18.0, 84.0] | 45.0 [18.0, 93.0] | 45.0 [18.0, 93.0] |
| Please indicate your gender. |  |  |  |
| Male | 354 (45.5%) | 378 (50.5%) | 732 (48.0%) |
| Female | 416 (53.5%) | 361 (48.3%) | 777 (50.9%) |
| Non-binary | 7 (0.9%) | 9 (1.2%) | 16 (1.0%) |
| Transgender | 1 (0.1%) | 0 (0%) | 1 (0.1%) |
| Please indicate your race. |  |  |  |
| White | 626 (80.5%) | 567 (75.8%) | 1193 (78.2%) |
| Black or African American | 99 (12.7%) | 108 (14.4%) | 207 (13.6%) |
| American Indian or Alaska Native | 3 (0.4%) | 1 (0.1%) | 4 (0.3%) |
| Asian | 40 (5.1%) | 54 (7.2%) | 94 (6.2%) |
| Native Hawaiian or Pacific Islander | 0 (0%) | 0 (0%) | 0 (0%) |
| Other | 10 (1.3%) | 18 (2.4%) | 28 (1.8%) |
| Are you of Hispanic or Latino origin? |  |  |  |
| Yes | 37 (4.8%) | 46 (6.1%) | 83 (5.4%) |
| No | 741 (95.2%) | 702 (93.9%) | 1443 (94.6%) |
| What is the highest grade or level of school you have completed, or the highest degree you have received? |  |  |  |
| Less than high school (i.e., no diploma or GED) | 9 (1.2%) | 7 (0.9%) | 16 (1.0%) |
| High school graduate, GED, or equivalent | 111 (14.3%) | 87 (11.6%) | 198 (13.0%) |
| Some college, but no degree | 167 (21.5%) | 163 (21.8%) | 330 (21.6%) |
| Associate degree or bachelor's degree (such as AA, AS, BA, BSW) | 341 (43.8%) | 354 (47.3%) | 695 (45.5%) |
| Master's degree (such as MA, MBA, MEd) | 104 (13.4%) | 106 (14.2%) | 210 (13.8%) |
| Doctoral or professional school degree (such as PhD, MD, JD) | 46 (5.9%) | 31 (4.1%) | 77 (5.0%) |
| How often do you read science papers or science in the news? |  |  |  |
| Mean (SD) | 2.93 (0.856) | 2.95 (0.917) | 2.94 (0.886) |
| Median [Min, Max] | 3.00 [1.00, 5.00] | 3.00 [1.00, 5.00] | 3.00 [1.00, 5.00] |
| Missing | 5 (0.6%) | 4 (0.5%) | 9 (0.6%) |
| Regarding your political orientation, would you consider yourself to be... |  |  |  |
| Mean (SD) | 2.05 (0.673) | 2.06 (0.673) | 2.05 (0.673) |
| Median [Min, Max] | 2.00 [1.00, 3.00] | 2.00 [1.00, 3.00] | 2.00 [1.00, 3.00] |
| Missing | 5 (0.6%) | 4 (0.5%) | 9 (0.6%) |
| How would you describe your political orientation? |  |  |  |
| Mean (SD) | 4.07 (2.72) | 4.11 (2.67) | 4.09 (2.70) |
| Median [Min, Max] | 3.00 [1.00, 10.0] | 4.00 [1.00, 10.0] | 4.00 [1.00, 10.0] |
| Missing | 4 (0.5%) | 4 (0.5%) | 8 (0.5%) |
| How would you describe your level of religious commitment (this refers to any religious belief system)? |  |  |  |
| Mean (SD) | 3.94 (3.38) | 3.76 (3.31) | 3.85 (3.35) |
| Median [Min, Max] | 2.00 [1.00, 10.0] | 2.00 [1.00, 10.0] | 2.00 [1.00, 10.0] |
| Missing | 4 (0.5%) | 4 (0.5%) | 8 (0.5%) |
| TrustTotal |  |  |  |
| Mean (SD) | 3.72 (0.695) | 3.72 (0.730) | 3.72 (0.712) |
| Median [Min, Max] | 3.81 [1.29, 5.00] | 3.86 [1.00, 5.00] | 3.81 [1.00, 5.00] |
| Missing | 1 (0.1%) | 1 (0.1%) | 2 (0.1%) |
| How credible is the scientist who conducted the study described in the post? |  |  |  |
| Mean (SD) | 4.96 (1.45) | 4.87 (1.47) | 4.92 (1.46) |
| Median [Min, Max] | 5.00 [1.00, 7.00] | 5.00 [1.00, 7.00] | 5.00 [1.00, 7.00] |
| How credible is the research described in the post? |  |  |  |
| Mean (SD) | 4.98 (1.57) | 4.93 (1.57) | 4.96 (1.57) |
| Median [Min, Max] | 5.00 [1.00, 7.00] | 5.00 [1.00, 7.00] | 5.00 [1.00, 7.00] |
| I would trust scientific information if I knew it came from this author. |  |  |  |
| Mean (SD) | 4.74 (1.47) | 4.70 (1.50) | 4.72 (1.49) |
| Median [Min, Max] | 5.00 [1.00, 7.00] | 5.00 [1.00, 7.00] | 5.00 [1.00, 7.00] |
| I trust this scientific information. |  |  |  |
| Mean (SD) | 5.11 (1.62) | 5.03 (1.62) | 5.07 (1.62) |
| Median [Min, Max] | 6.00 [1.00, 7.00] | 5.00 [1.00, 7.00] | 5.00 [1.00, 7.00] |

## Values as Categories

```
table1(~Age+Gender+Race+Ethnicity+Educ+Q57+Party+Political+Religious+TrustTotal+CredibleSci+CredibleRes+TrustAuth+TrustInfo|Arm,data=df_cat)
```

|  | 1 (N=778) | 2 (N=748) | Overall (N=1526) |
| --- | --- | --- | --- |
| About how old are you (in years)? |  |  |  |
| Mean (SD) | 45.8 (16.3) | 45.2 (15.8) | 45.5 (16.1) |
| Median [Min, Max] | 46.0 [18.0, 84.0] | 45.0 [18.0, 93.0] | 45.0 [18.0, 93.0] |
| Please indicate your gender. |  |  |  |
| Male | 354 (45.5%) | 378 (50.5%) | 732 (48.0%) |
| Female | 416 (53.5%) | 361 (48.3%) | 777 (50.9%) |
| Non-binary | 7 (0.9%) | 9 (1.2%) | 16 (1.0%) |
| Transgender | 1 (0.1%) | 0 (0%) | 1 (0.1%) |
| Please indicate your race. |  |  |  |
| White | 626 (80.5%) | 567 (75.8%) | 1193 (78.2%) |
| Black or African American | 99 (12.7%) | 108 (14.4%) | 207 (13.6%) |
| American Indian or Alaska Native | 3 (0.4%) | 1 (0.1%) | 4 (0.3%) |
| Asian | 40 (5.1%) | 54 (7.2%) | 94 (6.2%) |
| Native Hawaiian or Pacific Islander | 0 (0%) | 0 (0%) | 0 (0%) |
| Other | 10 (1.3%) | 18 (2.4%) | 28 (1.8%) |
| Are you of Hispanic or Latino origin? |  |  |  |
| Yes | 37 (4.8%) | 46 (6.1%) | 83 (5.4%) |
| No | 741 (95.2%) | 702 (93.9%) | 1443 (94.6%) |
| What is the highest grade or level of school you have completed, or the highest degree you have received? |  |  |  |
| Less than high school (i.e., no diploma or GED) | 9 (1.2%) | 7 (0.9%) | 16 (1.0%) |
| High school graduate, GED, or equivalent | 111 (14.3%) | 87 (11.6%) | 198 (13.0%) |
| Some college, but no degree | 167 (21.5%) | 163 (21.8%) | 330 (21.6%) |
| Associate degree or bachelor's degree (such as AA, AS, BA, BSW) | 341 (43.8%) | 354 (47.3%) | 695 (45.5%) |
| Master's degree (such as MA, MBA, MEd) | 104 (13.4%) | 106 (14.2%) | 210 (13.8%) |
| Doctoral or professional school degree (such as PhD, MD, JD) | 46 (5.9%) | 31 (4.1%) | 77 (5.0%) |
| How often do you read science papers or science in the news? |  |  |  |
| Never | 32 (4.1%) | 41 (5.5%) | 73 (4.8%) |
| Rarely | 191 (24.6%) | 177 (23.7%) | 368 (24.1%) |
| Sometimes | 377 (48.5%) | 331 (44.3%) | 708 (46.4%) |
| Very Often | 147 (18.9%) | 165 (22.1%) | 312 (20.4%) |
| Always | 26 (3.3%) | 30 (4.0%) | 56 (3.7%) |
| Missing | 5 (0.6%) | 4 (0.5%) | 9 (0.6%) |
| Regarding your political orientation, would you consider yourself to be... |  |  |  |
| Republican | 158 (20.3%) | 147 (19.7%) | 305 (20.0%) |
| Democrat | 422 (54.2%) | 405 (54.1%) | 827 (54.2%) |
| Other | 193 (24.8%) | 192 (25.7%) | 385 (25.2%) |
| Missing | 5 (0.6%) | 4 (0.5%) | 9 (0.6%) |
| How would you describe your political orientation? |  |  |  |
| 1 - Liberal | 181 (23.3%) | 161 (21.5%) | 342 (22.4%) |
| 2 | 101 (13.0%) | 102 (13.6%) | 203 (13.3%) |
| 3 | 107 (13.8%) | 89 (11.9%) | 196 (12.8%) |
| 4 | 60 (7.7%) | 75 (10.0%) | 135 (8.8%) |
| 5 | 112 (14.4%) | 131 (17.5%) | 243 (15.9%) |
| 6 | 69 (8.9%) | 47 (6.3%) | 116 (7.6%) |
| 7 | 34 (4.4%) | 34 (4.5%) | 68 (4.5%) |
| 8 | 38 (4.9%) | 41 (5.5%) | 79 (5.2%) |
| 9 | 28 (3.6%) | 20 (2.7%) | 48 (3.1%) |
| 10 - Conservative | 44 (5.7%) | 44 (5.9%) | 88 (5.8%) |
| Missing | 4 (0.5%) | 4 (0.5%) | 8 (0.5%) |
| How would you describe your level of religious commitment (this refers to any religious belief system)? |  |  |  |
| 1 - Low commitment | 341 (43.8%) | 336 (44.9%) | 677 (44.4%) |
| 2 | 69 (8.9%) | 81 (10.8%) | 150 (9.8%) |
| 3 | 45 (5.8%) | 46 (6.1%) | 91 (6.0%) |
| 4 | 28 (3.6%) | 21 (2.8%) | 49 (3.2%) |
| 5 | 26 (3.3%) | 25 (3.3%) | 51 (3.3%) |
| 6 | 42 (5.4%) | 26 (3.5%) | 68 (4.5%) |
| 7 | 48 (6.2%) | 47 (6.3%) | 95 (6.2%) |
| 8 | 46 (5.9%) | 58 (7.8%) | 104 (6.8%) |
| 9 | 50 (6.4%) | 39 (5.2%) | 89 (5.8%) |
| 10 - High commitment | 79 (10.2%) | 65 (8.7%) | 144 (9.4%) |
| Missing | 4 (0.5%) | 4 (0.5%) | 8 (0.5%) |
| TrustTotal |  |  |  |
| Mean (SD) | 3.72 (0.695) | 3.72 (0.730) | 3.72 (0.712) |
| Median [Min, Max] | 3.81 [1.29, 5.00] | 3.86 [1.00, 5.00] | 3.81 [1.00, 5.00] |
| Missing | 1 (0.1%) | 1 (0.1%) | 2 (0.1%) |
| How credible is the scientist who conducted the study described in the post? |  |  |  |
| 1 - Not at all credible | 22 (2.8%) | 24 (3.2%) | 46 (3.0%) |
| 2 | 28 (3.6%) | 31 (4.1%) | 59 (3.9%) |
| 3 | 51 (6.6%) | 39 (5.2%) | 90 (5.9%) |
| 4 | 181 (23.3%) | 203 (27.1%) | 384 (25.2%) |
| 5 | 183 (23.5%) | 189 (25.3%) | 372 (24.4%) |
| 6 | 199 (25.6%) | 150 (20.1%) | 349 (22.9%) |
| 7 - Extremely Credible | 114 (14.7%) | 112 (15.0%) | 226 (14.8%) |
| How credible is the research described in the post? |  |  |  |
| 1 - Not at all credible | 28 (3.6%) | 34 (4.5%) | 62 (4.1%) |
| 2 | 42 (5.4%) | 29 (3.9%) | 71 (4.7%) |
| 3 | 47 (6.0%) | 46 (6.1%) | 93 (6.1%) |
| 4 | 148 (19.0%) | 161 (21.5%) | 309 (20.2%) |
| 5 | 188 (24.2%) | 189 (25.3%) | 377 (24.7%) |
| 6 | 186 (23.9%) | 153 (20.5%) | 339 (22.2%) |
| 7 - Extremely credible | 139 (17.9%) | 136 (18.2%) | 275 (18.0%) |
| I would trust scientific information if I knew it came from this author. |  |  |  |
| 1 - Strongly Disagree | 31 (4.0%) | 33 (4.4%) | 64 (4.2%) |
| 2 - Disagree | 40 (5.1%) | 39 (5.2%) | 79 (5.2%) |
| 3 - Somewhat Disagree | 48 (6.2%) | 39 (5.2%) | 87 (5.7%) |
| 4 - Neither Agree nor Disagree | 203 (26.1%) | 220 (29.4%) | 423 (27.7%) |
| 5 - Somewhat Agree | 186 (23.9%) | 176 (23.5%) | 362 (23.7%) |
| 6 - Agree | 197 (25.3%) | 159 (21.3%) | 356 (23.3%) |
| 7 - Strongly Agree | 73 (9.4%) | 82 (11.0%) | 155 (10.2%) |
| I trust this scientific information. |  |  |  |
| 1 - Strongly Disagree | 33 (4.2%) | 35 (4.7%) | 68 (4.5%) |
| 2 - Disagree | 47 (6.0%) | 42 (5.6%) | 89 (5.8%) |
| 3 - Somewhat Disagree | 46 (5.9%) | 45 (6.0%) | 91 (6.0%) |
| 4 - Neither Agree nor Disagree | 88 (11.3%) | 99 (13.2%) | 187 (12.3%) |
| 5 - Somewhat Agree | 169 (21.7%) | 180 (24.1%) | 349 (22.9%) |
| 6 - Agree | 255 (32.8%) | 216 (28.9%) | 471 (30.9%) |
| 7 - Strongly Agree | 140 (18.0%) | 131 (17.5%) | 271 (17.8%) |

# Statistical analysis adjusted

```
# Define dependent variables
vars <- c("TrustTotal", "CredibleSci", "CredibleRes", "TrustAuth", "TrustInfo")
#n <- "TrustTotal"
for (n in vars) {
  
  # Title with dependent
  cat("  \n\n")
  cat("  \n## ",n,"  \n", sep="")
  
  # Model
  mod1 <- lm(df[[n]] ~ Arm+Age+Gender+Race+Ethnicity+Educ+Q57+Party+Political+Religious, data=df)
  
  # Model summary
  t1 <- tbl_regression(mod1, exp=FALSE, pvalue_fun=~style_pvalue(.x, digits = 3))

  # Type 3 table
  t2 <- kable(prettify(Anova(mod1, type=3)), digits = 3, 
            col.names = c("Variable", "Sum Sq", "Df", "F value", "p-value", "")) %>%
            kable_styling(full_width = FALSE)
  
  # Assumptions
  as1 <- cbind(R2 = summary(mod1)$r.squared,
              adjR2 = summary(mod1)$adj.r.squared,
              Skewness = skewness(resid(mod1)),
              LeveneTest_p = leveneTest(df[[n]]~Arm, df)["group","Pr(>F)"]
              ) 
  t3 <- kable(as1, digits=c(2,2,2,3), row.names = F) %>%
        kable_styling(bootstrap_options = "striped", full_width = F)
  
  # Plot
  p1 <- ggplot(df, aes(y=.data[[n]], x=Arm, fill=Arm))+
  geom_boxplot()+
  scale_fill_manual(values=c("#0072B2","#E69F00"))+
  stat_summary(fun="mean", shape=8)+
  theme_classic()
  
  
  
  # Model with interaction
  mod2 <- lm(df[[n]] ~ Arm*Political+Age+Gender+Race+Ethnicity+Educ+Q57+Party+Religious, data=df)
  
  # Model summary
  t4 <- tbl_regression(mod2, exp=FALSE, pvalue_fun=~style_pvalue(.x, digits = 3))

  # Type 3 table
  t5 <- kable(prettify(Anova(mod2, type=3)), digits = 3, 
            col.names = c("Variable", "Sum Sq", "Df", "F value", "p-value", "")) %>%
            kable_styling(full_width = FALSE)
  
  # Assumptions
  as2 <- cbind(R2 = summary(mod2)$r.squared,
              adjR2 = summary(mod2)$adj.r.squared,
              Skewness = skewness(resid(mod2)),
              LeveneTest_p = leveneTest(df[[n]]~Arm, df)["group","Pr(>F)"]
              ) 
  t6 <- kable(as2, digits=c(2,2,2,3), row.names = F) %>%
        kable_styling(bootstrap_options = "striped", full_width = F)
  
  p2 <- ggplot(df, aes(y=.data[[n]], x=Political, color=Arm, group=Arm))+
  geom_smooth(method = "lm")+
  scale_color_manual(values=c("#0072B2","#E69F00"))+
  theme_classic()
  
  
  # Print tables
  cat("  \n")
  cat("  \n### Only main effects{.tabset} \n")

  print(p1)

  cat("  \n")
  cat("  \n#### Type 3 table \n")
  cat("  \n Type 3 table \n", sep="")
  print(t2)

  cat("  \n")
  cat("  \n\n#### Estimates table \n")
  cat("  \n Estimates table  \n", sep="")
  print(t1)
  
  cat("  \n")
  cat("  \n\n#### Assumptions & Goodness-of-fit\n")
  cat("  \n $R^2$ is the variance explained by the model \n", sep="")
  cat("  \n To test for normality of residuals, we want the skewness of the residual to be between -2 and 2 \n", sep="")
  cat("  \n To test for equal variance, We want to have a non-significant p-value for Levene's Test \n", sep="")
  print(t3)
  
  cat("  \n")
  cat("  \n### Arm and political interaction{.tabset} \n")

  print(p2)

  cat("  \n")
  cat("  \n#### Type 3 table \n")
  cat("  \n Type 3 table \n", sep="")
  print(t5)

  cat("  \n")
  cat("  \n\n#### Estimates table \n")
  cat("  \n Estimates table  \n", sep="")
  print(t4)
  
  cat("  \n")
  cat("  \n\n#### Assumptions & Goodness-of-fit\n")
  cat("  \n $R^2$ is the variance explained by the model \n", sep="")
  cat("  \n To test for normality of residuals, we want the skewness of the residual to be between -2 and 2 \n", sep="")
  cat("  \n To test for equal variance, We want to have a non-significant p-value for Levene's Test \n", sep="")
  print(t6)
  
}
```

## TrustTotal

### Only main effects

#### Type 3 table

Type 3 table

| Variable | Sum Sq | Df | F value | p-value |  |
| --- | --- | --- | --- | --- | --- |
| (Intercept) | 153.091 | 1 | 458.819 | <0.001 | \*\*\* |
| Arm | 0.007 | 1 | 0.020 | 0.887 |  |
| Age | 0.819 | 1 | 2.455 | 0.117 |  |
| Gender | 3.219 | 3 | 3.215 | 0.022 |  |
| Race | 4.442 | 4 | 3.328 | 0.01 |  |
| Ethnicity | 0.695 | 1 | 2.082 | 0.149 |  |
| Educ | 4.535 | 5 | 2.718 | 0.019 |  |
| Q57 | 17.987 | 1 | 53.907 | <0.001 | \*\*\* |
| Party | 3.129 | 1 | 9.376 | 0.002 | \*\* |
| Political | 139.021 | 1 | 416.652 | <0.001 | \*\*\* |
| Religious | 1.076 | 1 | 3.225 | 0.073 | . |

#### Estimates table

Estimates table

#### Assumptions & Goodness-of-fit

\(R^2\) is the variance explained by
the model

To test for normality of residuals, we want the skewness of the
residual to be between -2 and 2

To test for equal variance, We want to have a non-significant p-value
for Levene’s Test

| R2 | adjR2 | Skewness | LeveneTest\_p |
| --- | --- | --- | --- |
| 0.34 | 0.33 | -0.38 | 0.585 |

### Arm and political interaction

#### Type 3 table

Type 3 table

| Variable | Sum Sq | Df | F value | p-value |  |
| --- | --- | --- | --- | --- | --- |
| (Intercept) | 150.577 | 1 | 450.990 | <0.001 | \*\*\* |
| Arm | 0.000 | 1 | 0.001 | 0.97 |  |
| Political | 84.225 | 1 | 252.260 | <0.001 | \*\*\* |
| Age | 0.814 | 1 | 2.439 | 0.119 |  |
| Gender | 3.220 | 3 | 3.215 | 0.022 |  |
| Race | 4.443 | 4 | 3.327 | 0.01 |  |
| Ethnicity | 0.698 | 1 | 2.092 | 0.148 |  |
| Educ | 4.522 | 5 | 2.709 | 0.019 |  |
| Q57 | 17.983 | 1 | 53.861 | <0.001 | \*\*\* |
| Party | 3.130 | 1 | 9.374 | 0.002 | \*\* |
| Religious | 1.075 | 1 | 3.220 | 0.073 | . |
| Arm:Political | 0.006 | 1 | 0.019 | 0.889 |  |

#### Estimates table

Estimates table

#### Assumptions & Goodness-of-fit

\(R^2\) is the variance explained by
the model

To test for normality of residuals, we want the skewness of the
residual to be between -2 and 2

To test for equal variance, We want to have a non-significant p-value
for Levene’s Test

| R2 | adjR2 | Skewness | LeveneTest\_p |
| --- | --- | --- | --- |
| 0.34 | 0.33 | -0.38 | 0.585 |

## CredibleSci

### Only main effects

#### Type 3 table

Type 3 table

| Variable | Sum Sq | Df | F value | p-value |  |
| --- | --- | --- | --- | --- | --- |
| (Intercept) | 193.653 | 1 | 110.421 | <0.001 | \*\*\* |
| Arm | 2.276 | 1 | 1.298 | 0.255 |  |
| Age | 20.241 | 1 | 11.542 | 0.001 | \*\*\* |
| Gender | 4.616 | 3 | 0.877 | 0.452 |  |
| Race | 9.509 | 4 | 1.355 | 0.247 |  |
| Ethnicity | 1.060 | 1 | 0.604 | 0.437 |  |
| Educ | 17.134 | 5 | 1.954 | 0.083 | . |
| Q57 | 40.175 | 1 | 22.908 | <0.001 | \*\*\* |
| Party | 5.906 | 1 | 3.368 | 0.067 | . |
| Political | 426.292 | 1 | 243.073 | <0.001 | \*\*\* |
| Religious | 15.268 | 1 | 8.706 | 0.003 | \*\* |

#### Estimates table

Estimates table

#### Assumptions & Goodness-of-fit

\(R^2\) is the variance explained by
the model

To test for normality of residuals, we want the skewness of the
residual to be between -2 and 2

To test for equal variance, We want to have a non-significant p-value
for Levene’s Test

| R2 | adjR2 | Skewness | LeveneTest\_p |
| --- | --- | --- | --- |
| 0.19 | 0.18 | -0.42 | 0.911 |

### Arm and political interaction

#### Type 3 table

Type 3 table

| Variable | Sum Sq | Df | F value | p-value |  |
| --- | --- | --- | --- | --- | --- |
| (Intercept) | 197.512 | 1 | 112.715 | <0.001 | \*\*\* |
| Arm | 6.182 | 1 | 3.528 | 0.061 | . |
| Political | 302.512 | 1 | 172.636 | <0.001 | \*\*\* |
| Age | 19.732 | 1 | 11.260 | 0.001 | \*\*\* |
| Gender | 4.622 | 3 | 0.879 | 0.451 |  |
| Race | 9.912 | 4 | 1.414 | 0.227 |  |
| Ethnicity | 0.924 | 1 | 0.527 | 0.468 |  |
| Educ | 17.370 | 5 | 1.983 | 0.078 | . |
| Q57 | 40.293 | 1 | 22.994 | <0.001 | \*\*\* |
| Party | 5.863 | 1 | 3.346 | 0.068 | . |
| Religious | 15.171 | 1 | 8.658 | 0.003 | \*\* |
| Arm:Political | 3.933 | 1 | 2.244 | 0.134 |  |

#### Estimates table

Estimates table

#### Assumptions & Goodness-of-fit

\(R^2\) is the variance explained by
the model

To test for normality of residuals, we want the skewness of the
residual to be between -2 and 2

To test for equal variance, We want to have a non-significant p-value
for Levene’s Test

| R2 | adjR2 | Skewness | LeveneTest\_p |
| --- | --- | --- | --- |
| 0.19 | 0.18 | -0.42 | 0.911 |

## CredibleRes

### Only main effects

#### Type 3 table

Type 3 table

| Variable | Sum Sq | Df | F value | p-value |  |
| --- | --- | --- | --- | --- | --- |
| (Intercept) | 202.155 | 1 | 102.431 | <0.001 | \*\*\* |
| Arm | 0.279 | 1 | 0.141 | 0.707 |  |
| Age | 7.595 | 1 | 3.849 | 0.05 |  |
| Gender | 2.736 | 3 | 0.462 | 0.709 |  |
| Race | 9.404 | 4 | 1.191 | 0.313 |  |
| Ethnicity | 1.004 | 1 | 0.509 | 0.476 |  |
| Educ | 26.141 | 5 | 2.649 | 0.022 |  |
| Q57 | 43.589 | 1 | 22.086 | <0.001 | \*\*\* |
| Party | 5.086 | 1 | 2.577 | 0.109 |  |
| Political | 547.877 | 1 | 277.606 | <0.001 | \*\*\* |
| Religious | 16.374 | 1 | 8.297 | 0.004 | \*\* |

#### Estimates table

Estimates table

#### Assumptions & Goodness-of-fit

\(R^2\) is the variance explained by
the model

To test for normality of residuals, we want the skewness of the
residual to be between -2 and 2

To test for equal variance, We want to have a non-significant p-value
for Levene’s Test

| R2 | adjR2 | Skewness | LeveneTest\_p |
| --- | --- | --- | --- |
| 0.21 | 0.2 | -0.45 | 0.863 |

### Arm and political interaction

#### Type 3 table

Type 3 table

| Variable | Sum Sq | Df | F value | p-value |  |
| --- | --- | --- | --- | --- | --- |
| (Intercept) | 205.880 | 1 | 104.381 | <0.001 | \*\*\* |
| Arm | 3.644 | 1 | 1.847 | 0.174 |  |
| Political | 381.255 | 1 | 193.297 | <0.001 | \*\*\* |
| Age | 7.295 | 1 | 3.698 | 0.055 | . |
| Gender | 2.750 | 3 | 0.465 | 0.707 |  |
| Race | 9.837 | 4 | 1.247 | 0.289 |  |
| Ethnicity | 0.875 | 1 | 0.444 | 0.506 |  |
| Educ | 26.691 | 5 | 2.706 | 0.019 |  |
| Q57 | 43.709 | 1 | 22.160 | <0.001 | \*\*\* |
| Party | 5.047 | 1 | 2.559 | 0.11 |  |
| Religious | 16.275 | 1 | 8.252 | 0.004 | \*\* |
| Arm:Political | 3.762 | 1 | 1.907 | 0.167 |  |

#### Estimates table

Estimates table

#### Assumptions & Goodness-of-fit

\(R^2\) is the variance explained by
the model

To test for normality of residuals, we want the skewness of the
residual to be between -2 and 2

To test for equal variance, We want to have a non-significant p-value
for Levene’s Test

| R2 | adjR2 | Skewness | LeveneTest\_p |
| --- | --- | --- | --- |
| 0.21 | 0.2 | -0.45 | 0.863 |

## TrustAuth

### Only main effects

#### Type 3 table

Type 3 table

| Variable | Sum Sq | Df | F value | p-value |  |
| --- | --- | --- | --- | --- | --- |
| (Intercept) | 206.471 | 1 | 113.989 | <0.001 | \*\*\* |
| Arm | 0.585 | 1 | 0.323 | 0.57 |  |
| Age | 0.817 | 1 | 0.451 | 0.502 |  |
| Gender | 4.247 | 3 | 0.782 | 0.504 |  |
| Race | 7.640 | 4 | 1.055 | 0.378 |  |
| Ethnicity | 0.311 | 1 | 0.172 | 0.678 |  |
| Educ | 14.799 | 5 | 1.634 | 0.148 |  |
| Q57 | 45.668 | 1 | 25.213 | <0.001 | \*\*\* |
| Party | 3.817 | 1 | 2.107 | 0.147 |  |
| Political | 452.415 | 1 | 249.772 | <0.001 | \*\*\* |
| Religious | 16.187 | 1 | 8.936 | 0.003 | \*\* |

#### Estimates table

Estimates table  

| **Characteristic** | **Beta** | **95% CI**1 | **p-value** |
| --- | --- | --- | --- |
| Arm |  |  |  |
| 1 | — | — |  |
| 2 | -0.04 | -0.18, 0.10 | 0.570 |
| About how old are you (in years)? | 0.00 | 0.00, 0.01 | 0.502 |
| Please indicate your gender. |  |  |  |
| Male | — | — |  |
| Female | -0.09 | -0.23, 0.05 | 0.206 |
| Non-binary | -0.14 | -0.81, 0.54 | 0.689 |
| Transgender | -1.1 | -3.8, 1.5 | 0.401 |
| Race |  |  |  |
| White | — | — |  |
| Black or African American | 0.13 | -0.08, 0.34 | 0.237 |
| American Indian or Alaska Native | 1.1 | -0.23, 2.4 | 0.105 |
| Asian | 0.10 | -0.19, 0.39 | 0.501 |
| Other | 0.05 | -0.49, 0.58 | 0.868 |
| Are you of Hispanic or Latino origin? |  |  |  |
| Yes | — | — |  |
| No | 0.07 | -0.25, 0.39 | 0.678 |
| What is the highest grade or level of school you have completed, or the highest degree you have received? |  |  |  |
| Less than high school (i.e., no diploma or GED) | — | — |  |
| High school graduate, GED, or equivalent | 0.63 | -0.06, 1.3 | 0.073 |
| Some college, but no degree | 0.40 | -0.27, 1.1 | 0.243 |
| Associate degree or bachelor’s degree (such as AA, AS, BA, BSW) | 0.59 | -0.08, 1.3 | 0.083 |
| Master’s degree (such as MA, MBA, MEd) | 0.54 | -0.15, 1.2 | 0.124 |
| Doctoral or professional school degree (such as PhD, MD, JD) | 0.44 | -0.30, 1.2 | 0.245 |
| How often do you read science papers or science in the news? | 0.21 | 0.13, 0.29 | <0.001 |
| Regarding your political orientation, would you consider yourself to be… | -0.08 | -0.19, 0.03 | 0.147 |
| How would you describe your political orientation? | -0.24 | -0.27, -0.21 | <0.001 |
| How would you describe your level of religious commitment (this refers to any religious belief system)? | 0.04 | 0.01, 0.06 | 0.003 |
|  |  |  |  |
| --- | --- | --- | --- |
| 1 CI = Confidence Interval | | | |

#### Assumptions & Goodness-of-fit

\(R^2\) is the variance explained by
the model

To test for normality of residuals, we want the skewness of the
residual to be between -2 and 2

To test for equal variance, We want to have a non-significant p-value
for Levene’s Test

| R2 | adjR2 | Skewness | LeveneTest\_p |
| --- | --- | --- | --- |
| 0.19 | 0.18 | -0.37 | 0.628 |

### Arm and political interaction

#### Type 3 table

Type 3 table

| Variable | Sum Sq | Df | F value | p-value |  |
| --- | --- | --- | --- | --- | --- |
| (Intercept) | 213.207 | 1 | 117.968 | <0.001 | \*\*\* |
| Arm | 7.540 | 1 | 4.172 | 0.041 |  |
| Political | 337.860 | 1 | 186.938 | <0.001 | \*\*\* |
| Age | 0.682 | 1 | 0.377 | 0.539 |  |
| Gender | 4.134 | 3 | 0.762 | 0.515 |  |
| Race | 7.888 | 4 | 1.091 | 0.359 |  |
| Ethnicity | 0.214 | 1 | 0.118 | 0.731 |  |
| Educ | 14.800 | 5 | 1.638 | 0.147 |  |
| Q57 | 45.844 | 1 | 25.366 | <0.001 | \*\*\* |
| Party | 3.768 | 1 | 2.085 | 0.149 |  |
| Religious | 16.046 | 1 | 8.878 | 0.003 | \*\* |
| Arm:Political | 7.767 | 1 | 4.297 | 0.038 |  |

#### Estimates table

Estimates table  

| **Characteristic** | **Beta** | **95% CI**1 | **p-value** |
| --- | --- | --- | --- |
| Arm |  |  |  |
| 1 | — | — |  |
| 2 | -0.26 | -0.50, -0.01 | 0.041 |
| How would you describe your political orientation? | -0.27 | -0.31, -0.23 | <0.001 |
| About how old are you (in years)? | 0.00 | 0.00, 0.01 | 0.539 |
| Please indicate your gender. |  |  |  |
| Male | — | — |  |
| Female | -0.09 | -0.23, 0.05 | 0.208 |
| Non-binary | -0.13 | -0.80, 0.55 | 0.711 |
| Transgender | -1.1 | -3.8, 1.5 | 0.410 |
| Race |  |  |  |
| White | — | — |  |
| Black or African American | 0.13 | -0.09, 0.34 | 0.245 |
| American Indian or Alaska Native | 1.2 | -0.18, 2.5 | 0.089 |
| Asian | 0.09 | -0.20, 0.38 | 0.546 |
| Other | 0.03 | -0.50, 0.56 | 0.915 |
| Are you of Hispanic or Latino origin? |  |  |  |
| Yes | — | — |  |
| No | 0.06 | -0.26, 0.38 | 0.731 |
| What is the highest grade or level of school you have completed, or the highest degree you have received? |  |  |  |
| Less than high school (i.e., no diploma or GED) | — | — |  |
| High school graduate, GED, or equivalent | 0.65 | -0.04, 1.3 | 0.065 |
| Some college, but no degree | 0.42 | -0.26, 1.1 | 0.227 |
| Associate degree or bachelor’s degree (such as AA, AS, BA, BSW) | 0.60 | -0.07, 1.3 | 0.078 |
| Master’s degree (such as MA, MBA, MEd) | 0.56 | -0.13, 1.2 | 0.113 |
| Doctoral or professional school degree (such as PhD, MD, JD) | 0.45 | -0.28, 1.2 | 0.225 |
| How often do you read science papers or science in the news? | 0.21 | 0.13, 0.29 | <0.001 |
| Regarding your political orientation, would you consider yourself to be… | -0.08 | -0.19, 0.03 | 0.149 |
| How would you describe your level of religious commitment (this refers to any religious belief system)? | 0.04 | 0.01, 0.06 | 0.003 |
| Arm \* How would you describe your political orientation? |  |  |  |
| 2 \* How would you describe your political orientation? | 0.05 | 0.00, 0.10 | 0.038 |
|  |  |  |  |
| --- | --- | --- | --- |
| 1 CI = Confidence Interval | | | |

#### Assumptions & Goodness-of-fit

\(R^2\) is the variance explained by
the model

To test for normality of residuals, we want the skewness of the
residual to be between -2 and 2

To test for equal variance, We want to have a non-significant p-value
for Levene’s Test

| R2 | adjR2 | Skewness | LeveneTest\_p |
| --- | --- | --- | --- |
| 0.19 | 0.18 | -0.37 | 0.628 |

## TrustInfo

### Only main effects

#### Type 3 table

Type 3 table

| Variable | Sum Sq | Df | F value | p-value |  |
| --- | --- | --- | --- | --- | --- |
| (Intercept) | 224.166 | 1 | 115.752 | <0.001 | \*\*\* |
| Arm | 1.239 | 1 | 0.640 | 0.424 |  |
| Age | 0.111 | 1 | 0.057 | 0.811 |  |
| Gender | 4.400 | 3 | 0.757 | 0.518 |  |
| Race | 5.356 | 4 | 0.691 | 0.598 |  |
| Ethnicity | 4.051 | 1 | 2.092 | 0.148 |  |
| Educ | 22.987 | 5 | 2.374 | 0.037 |  |
| Q57 | 52.262 | 1 | 26.986 | <0.001 | \*\*\* |
| Party | 2.289 | 1 | 1.182 | 0.277 |  |
| Political | 728.699 | 1 | 376.277 | <0.001 | \*\*\* |
| Religious | 11.661 | 1 | 6.021 | 0.014 |  |

#### Estimates table

Estimates table  

| **Characteristic** | **Beta** | **95% CI**1 | **p-value** |
| --- | --- | --- | --- |
| Arm |  |  |  |
| 1 | — | — |  |
| 2 | -0.06 | -0.20, 0.08 | 0.424 |
| About how old are you (in years)? | 0.00 | -0.01, 0.00 | 0.811 |
| Please indicate your gender. |  |  |  |
| Male | — | — |  |
| Female | -0.06 | -0.21, 0.08 | 0.390 |
| Non-binary | 0.15 | -0.55, 0.85 | 0.671 |
| Transgender | 1.6 | -1.2, 4.3 | 0.269 |
| Race |  |  |  |
| White | — | — |  |
| Black or African American | 0.13 | -0.09, 0.35 | 0.240 |
| American Indian or Alaska Native | 0.38 | -1.0, 1.8 | 0.589 |
| Asian | -0.04 | -0.34, 0.26 | 0.802 |
| Other | -0.26 | -0.81, 0.29 | 0.354 |
| Are you of Hispanic or Latino origin? |  |  |  |
| Yes | — | — |  |
| No | 0.24 | -0.09, 0.57 | 0.148 |
| What is the highest grade or level of school you have completed, or the highest degree you have received? |  |  |  |
| Less than high school (i.e., no diploma or GED) | — | — |  |
| High school graduate, GED, or equivalent | 1.0 | 0.31, 1.7 | 0.005 |
| Some college, but no degree | 0.72 | 0.02, 1.4 | 0.043 |
| Associate degree or bachelor’s degree (such as AA, AS, BA, BSW) | 0.85 | 0.16, 1.5 | 0.016 |
| Master’s degree (such as MA, MBA, MEd) | 0.88 | 0.16, 1.6 | 0.016 |
| Doctoral or professional school degree (such as PhD, MD, JD) | 0.76 | 0.00, 1.5 | 0.049 |
| How often do you read science papers or science in the news? | 0.22 | 0.14, 0.31 | <0.001 |
| Regarding your political orientation, would you consider yourself to be… | -0.06 | -0.18, 0.05 | 0.277 |
| How would you describe your political orientation? | -0.31 | -0.34, -0.28 | <0.001 |
| How would you describe your level of religious commitment (this refers to any religious belief system)? | 0.03 | 0.01, 0.05 | 0.014 |
|  |  |  |  |
| --- | --- | --- | --- |
| 1 CI = Confidence Interval | | | |

#### Assumptions & Goodness-of-fit

\(R^2\) is the variance explained by
the model

To test for normality of residuals, we want the skewness of the
residual to be between -2 and 2

To test for equal variance, We want to have a non-significant p-value
for Levene’s Test

| R2 | adjR2 | Skewness | LeveneTest\_p |
| --- | --- | --- | --- |
| 0.27 | 0.26 | -0.6 | 0.908 |

### Arm and political interaction

#### Type 3 table

Type 3 table

| Variable | Sum Sq | Df | F value | p-value |  |
| --- | --- | --- | --- | --- | --- |
| (Intercept) | 227.416 | 1 | 117.483 | <0.001 | \*\*\* |
| Arm | 4.485 | 1 | 2.317 | 0.128 |  |
| Political | 494.988 | 1 | 255.712 | <0.001 | \*\*\* |
| Age | 0.146 | 1 | 0.076 | 0.783 |  |
| Gender | 4.467 | 3 | 0.769 | 0.511 |  |
| Race | 5.603 | 4 | 0.724 | 0.576 |  |
| Ethnicity | 3.803 | 1 | 1.965 | 0.161 |  |
| Educ | 23.371 | 5 | 2.415 | 0.034 |  |
| Q57 | 52.384 | 1 | 27.062 | <0.001 | \*\*\* |
| Party | 2.265 | 1 | 1.170 | 0.28 |  |
| Religious | 11.584 | 1 | 5.984 | 0.015 |  |
| Arm:Political | 3.250 | 1 | 1.679 | 0.195 |  |

#### Estimates table

Estimates table  

| **Characteristic** | **Beta** | **95% CI**1 | **p-value** |
| --- | --- | --- | --- |
| Arm |  |  |  |
| 1 | — | — |  |
| 2 | -0.20 | -0.45, 0.06 | 0.128 |
| How would you describe your political orientation? | -0.33 | -0.37, -0.29 | <0.001 |
| About how old are you (in years)? | 0.00 | -0.01, 0.00 | 0.783 |
| Please indicate your gender. |  |  |  |
| Male | — | — |  |
| Female | -0.06 | -0.21, 0.08 | 0.393 |
| Non-binary | 0.16 | -0.54, 0.86 | 0.657 |
| Transgender | 1.6 | -1.2, 4.3 | 0.264 |
| Race |  |  |  |
| White | — | — |  |
| Black or African American | 0.13 | -0.09, 0.35 | 0.245 |
| American Indian or Alaska Native | 0.41 | -0.96, 1.8 | 0.556 |
| Asian | -0.04 | -0.35, 0.26 | 0.769 |
| Other | -0.27 | -0.82, 0.28 | 0.335 |
| Are you of Hispanic or Latino origin? |  |  |  |
| Yes | — | — |  |
| No | 0.24 | -0.09, 0.57 | 0.161 |
| What is the highest grade or level of school you have completed, or the highest degree you have received? |  |  |  |
| Less than high school (i.e., no diploma or GED) | — | — |  |
| High school graduate, GED, or equivalent | 1.0 | 0.32, 1.7 | 0.004 |
| Some college, but no degree | 0.73 | 0.03, 1.4 | 0.041 |
| Associate degree or bachelor’s degree (such as AA, AS, BA, BSW) | 0.86 | 0.16, 1.6 | 0.016 |
| Master’s degree (such as MA, MBA, MEd) | 0.89 | 0.17, 1.6 | 0.015 |
| Doctoral or professional school degree (such as PhD, MD, JD) | 0.77 | 0.01, 1.5 | 0.046 |
| How often do you read science papers or science in the news? | 0.22 | 0.14, 0.31 | <0.001 |
| Regarding your political orientation, would you consider yourself to be… | -0.06 | -0.18, 0.05 | 0.280 |
| How would you describe your level of religious commitment (this refers to any religious belief system)? | 0.03 | 0.01, 0.05 | 0.015 |
| Arm \* How would you describe your political orientation? |  |  |  |
| 2 \* How would you describe your political orientation? | 0.03 | -0.02, 0.09 | 0.195 |
|  |  |  |  |
| --- | --- | --- | --- |
| 1 CI = Confidence Interval | | | |

#### Assumptions & Goodness-of-fit

\(R^2\) is the variance explained by
the model

To test for normality of residuals, we want the skewness of the
residual to be between -2 and 2

To test for equal variance, We want to have a non-significant p-value
for Levene’s Test

| R2 | adjR2 | Skewness | LeveneTest\_p |
| --- | --- | --- | --- |
| 0.27 | 0.26 | -0.6 | 0.908 |

# Statistical analysis unadjusted

```
# Define dependent variables
vars <- c("TrustTotal", "CredibleSci", "CredibleRes", "TrustAuth", "TrustInfo")
#n <- "TrustTotal"
for (n in vars) {
  
  # Title with dependent
  cat("  \n\n")
  cat("  \n## ",n,"{.tabset}  \n", sep="")
  
  # Model
  mod1 <- lm(df[[n]] ~ Arm, data=df)
  
  # Model summary
  t1 <- tbl_regression(mod1, exp=FALSE, pvalue_fun=~style_pvalue(.x, digits = 3))

  # Type 3 table
  t2 <- kable(prettify(Anova(mod1, type=3)), digits = 3, 
            col.names = c("Variable", "Sum Sq", "Df", "F value", "p-value", "")) %>%
            kable_styling(full_width = FALSE)
  
  # Assumptions
  as1 <- cbind(R2 = summary(mod1)$r.squared,
              adjR2 = summary(mod1)$adj.r.squared,
              Skewness = skewness(resid(mod1)),
              LeveneTest_p = leveneTest(df[[n]]~Arm, df)["group","Pr(>F)"]
              ) 
  t3 <- kable(as1, digits=c(2,2,2,3), row.names = F) %>%
        kable_styling(bootstrap_options = "striped", full_width = F)
  
  # Plot
  p1 <- ggplot(df, aes(y=.data[[n]], x=Arm, fill=Arm))+
  geom_boxplot()+
  scale_fill_manual(values=c("#0072B2","#E69F00"))+
  stat_summary(fun="mean", shape=8)+
  theme_classic()
  
  
  
  # # Model with interaction
  # mod2 <- lm(df[[n]] ~ Arm, data=df)
  # 
  # # Model summary
  # t4 <- tbl_regression(mod2, exp=FALSE, pvalue_fun=~style_pvalue(.x, digits = 3))
  # 
  # # Type 3 table
  # t5 <- kable(prettify(Anova(mod2, type=3)), digits = 3, 
  #           col.names = c("Variable", "Sum Sq", "Df", "F value", "p-value", "")) %>%
  #           kable_styling(full_width = FALSE)
  # 
  # # Assumptions
  # as2 <- cbind(R2 = summary(mod2)$r.squared,
  #             adjR2 = summary(mod2)$adj.r.squared,
  #             Skewness = skewness(resid(mod2)),
  #             LeveneTest_p = leveneTest(df[[n]]~Arm, df)["group","Pr(>F)"]
  #             ) 
  # t6 <- kable(as2, digits=c(2,2,2,3), row.names = F) %>%
  #       kable_styling(bootstrap_options = "striped", full_width = F)
  # 
  # p2 <- ggplot(df, aes(y=.data[[n]], x=Political, color=Arm, group=Arm))+
  # geom_smooth(method = "lm")+
  # scale_color_manual(values=c("#0072B2","#E69F00"))+
  # theme_classic()
  
  
  # Print tables
  cat("  \n")
 # cat("  \n### Only main effects{.tabset} \n")

  print(p1)

  cat("  \n")
  cat("  \n### Type 3 table \n")
  cat("  \n Type 3 table \n", sep="")
  print(t2)

  cat("  \n")
  cat("  \n\n### Estimates table \n")
  cat("  \n Estimates table  \n", sep="")
  print(t1)
  
  cat("  \n")
  cat("  \n\n### Assumptions & Goodness-of-fit\n")
  cat("  \n $R^2$ is the variance explained by the model \n", sep="")
  cat("  \n To test for normality of residuals, we want the skewness of the residual to be between -2 and 2 \n", sep="")
  cat("  \n To test for equal variance, We want to have a non-significant p-value for Levene's Test \n", sep="")
  print(t3)
  
  cat("  \n")
  # cat("  \n### Arm and political interaction{.tabset} \n")
  # 
  # print(p2)
  # 
  # cat("  \n")
  # cat("  \n#### Type 3 table \n")
  # cat("  \n Type 3 table \n", sep="")
  # print(t5)
  # 
  # cat("  \n")
  # cat("  \n\n#### Estimates table \n")
  # cat("  \n Estimates table  \n", sep="")
  # print(t4)
  # 
  # cat("  \n")
  # cat("  \n\n#### Assumptions & Goodness-of-fit\n")
  # cat("  \n $R^2$ is the variance explained by the model \n", sep="")
  # cat("  \n To test for normality of residuals, we want the skewness of the residual to be between -2 and 2 \n", sep="")
  # cat("  \n To test for equal variance, We want to have a non-significant p-value for Levene's Test \n", sep="")
  # print(t6)
  
}
```

## TrustTotal

### Type 3 table

Type 3 table

| Variable | Sum Sq | Df | F value | p-value |  |
| --- | --- | --- | --- | --- | --- |
| (Intercept) | 10761.565 | 1 | 21213.960 | <0.001 | \*\*\* |
| Arm | 0.001 | 1 | 0.001 | 0.97 |  |

### Estimates table

Estimates table  

| **Characteristic** | **Beta** | **95% CI**1 | **p-value** |
| --- | --- | --- | --- |
| Arm |  |  |  |
| 1 | — | — |  |
| 2 | 0.00 | -0.07, 0.07 | 0.970 |
|  |  |  |  |
| --- | --- | --- | --- |
| 1 CI = Confidence Interval | | | |

### Assumptions & Goodness-of-fit

\(R^2\) is the variance explained by
the model

To test for normality of residuals, we want the skewness of the
residual to be between -2 and 2

To test for equal variance, We want to have a non-significant p-value
for Levene’s Test

| R2 | adjR2 | Skewness | LeveneTest\_p |
| --- | --- | --- | --- |
| 0 | 0 | -0.69 | 0.585 |

## CredibleSci

### Type 3 table

Type 3 table

| Variable | Sum Sq | Df | F value | p-value |  |
| --- | --- | --- | --- | --- | --- |
| (Intercept) | 19171.008 | 1 | 8987.871 | <0.001 | \*\*\* |
| Arm | 3.253 | 1 | 1.525 | 0.217 |  |

### Estimates table

Estimates table  

| **Characteristic** | **Beta** | **95% CI**1 | **p-value** |
| --- | --- | --- | --- |
| Arm |  |  |  |
| 1 | — | — |  |
| 2 | -0.09 | -0.24, 0.05 | 0.217 |
|  |  |  |  |
| --- | --- | --- | --- |
| 1 CI = Confidence Interval | | | |

### Assumptions & Goodness-of-fit

\(R^2\) is the variance explained by
the model

To test for normality of residuals, we want the skewness of the
residual to be between -2 and 2

To test for equal variance, We want to have a non-significant p-value
for Levene’s Test

| R2 | adjR2 | Skewness | LeveneTest\_p |
| --- | --- | --- | --- |
| 0 | 0 | -0.57 | 0.911 |

## CredibleRes

### Type 3 table

Type 3 table

| Variable | Sum Sq | Df | F value | p-value |  |
| --- | --- | --- | --- | --- | --- |
| (Intercept) | 19290.329 | 1 | 7845.461 | <0.001 | \*\*\* |
| Arm | 0.865 | 1 | 0.352 | 0.553 |  |

### Estimates table

Estimates table  

| **Characteristic** | **Beta** | **95% CI**1 | **p-value** |
| --- | --- | --- | --- |
| Arm |  |  |  |
| 1 | — | — |  |
| 2 | -0.05 | -0.21, 0.11 | 0.553 |
|  |  |  |  |
| --- | --- | --- | --- |
| 1 CI = Confidence Interval | | | |

### Assumptions & Goodness-of-fit

\(R^2\) is the variance explained by
the model

To test for normality of residuals, we want the skewness of the
residual to be between -2 and 2

To test for equal variance, We want to have a non-significant p-value
for Levene’s Test

| R2 | adjR2 | Skewness | LeveneTest\_p |
| --- | --- | --- | --- |
| 0 | 0 | -0.66 | 0.863 |

## TrustAuth

### Type 3 table

Type 3 table

| Variable | Sum Sq | Df | F value | p-value |  |
| --- | --- | --- | --- | --- | --- |
| (Intercept) | 17501.414 | 1 | 7929.867 | <0.001 | \*\*\* |
| Arm | 0.685 | 1 | 0.311 | 0.577 |  |

### Estimates table

Estimates table  

| **Characteristic** | **Beta** | **95% CI**1 | **p-value** |
| --- | --- | --- | --- |
| Arm |  |  |  |
| 1 | — | — |  |
| 2 | -0.04 | -0.19, 0.11 | 0.577 |
|  |  |  |  |
| --- | --- | --- | --- |
| 1 CI = Confidence Interval | | | |

### Assumptions & Goodness-of-fit

\(R^2\) is the variance explained by
the model

To test for normality of residuals, we want the skewness of the
residual to be between -2 and 2

To test for equal variance, We want to have a non-significant p-value
for Levene’s Test

| R2 | adjR2 | Skewness | LeveneTest\_p |
| --- | --- | --- | --- |
| 0 | 0 | -0.58 | 0.628 |

## TrustInfo

### Type 3 table

Type 3 table

| Variable | Sum Sq | Df | F value | p-value |  |
| --- | --- | --- | --- | --- | --- |
| (Intercept) | 20278.643 | 1 | 7753.980 | <0.001 | \*\*\* |
| Arm | 2.125 | 1 | 0.813 | 0.367 |  |

### Estimates table

Estimates table  

| **Characteristic** | **Beta** | **95% CI**1 | **p-value** |
| --- | --- | --- | --- |
| Arm |  |  |  |
| 1 | — | — |  |
| 2 | -0.07 | -0.24, 0.09 | 0.367 |
|  |  |  |  |
| --- | --- | --- | --- |
| 1 CI = Confidence Interval | | | |

### Assumptions & Goodness-of-fit

\(R^2\) is the variance explained by
the model

To test for normality of residuals, we want the skewness of the
residual to be between -2 and 2

To test for equal variance, We want to have a non-significant p-value
for Levene’s Test

| R2 | adjR2 | Skewness | LeveneTest\_p |
| --- | --- | --- | --- |
| 0 | 0 | -0.91 | 0.908 |
